# Disruption of cholesterol homeostasis triggers periodontal inflammation and alveolar bone loss

**DOI:** 10.1038/s12276-023-01122-w

**Published:** 2023-12-01

**Authors:** Thanh-Tam Tran, Gyuseok Lee, Yun Hyun Huh, Ki-Ho Chung, Sun Young Lee, Ka Hyon Park, Seung Hee Kwon, Min-Suk Kook, Jang-Soo Chun, Jeong-Tae Koh, Je-Hwang Ryu

**Affiliations:** 1 Department of Pharmacology and Dental Therapeutics, School of Dentistry, Chonnam National University, Gwangju, Korea; 2 Hard-tissue Biointerface Research Center, School of Dentistry, Chonnam National University, Gwangju, Korea; 3 School of Life Sciences, Gwangju Institute of Science and Technology, Gwangju, Korea; 4 Department of Preventive and Public Health Dentistry, School of Dentistry, Chonnam National University, Gwangju, Korea; 5 Department of Oral and Maxillofacial Surgery, School of Dentistry, Chonnam National University, Gwangju, Korea

**Keywords:** Mechanisms of disease, Translational research

## Abstract

Oral diseases exhibit a significant association with metabolic syndrome, including dyslipidemia. However, direct evidence supporting this relationship is lacking, and the involvement of cholesterol metabolism in the pathogenesis of periodontitis (PD) has yet to be determined. In this study, we showed that high cholesterol caused periodontal inflammation in mice. Cholesterol homeostasis in human gingival fibroblasts was disrupted by enhanced uptake through C-X-C motif chemokine ligand 16 (CXCL16), upregulation of cholesterol hydroxylase (CH25H), and the production of 25-hydroxycholesterol (an oxysterol metabolite of CH25H). Retinoid-related orphan receptor α (RORα) mediated the transcriptional upregulation of inflammatory mediators; consequently, PD pathogenesis mechanisms, including alveolar bone loss, were stimulated. Our collective data provided direct evidence that hyperlipidemia is a risk factor for PD and supported that inhibition of the CXCL16-CH25H-RORα axis is a potential treatment mechanism for PD as a systemic disorder manifestation.

## Cholesterol disruption: the hidden trigger for periodontal inflammation

Hypercholesterolemia, a type of hyperlipidemia, has been identified as a key factor in the development of periodontitis. This groundbreaking study by Tran et al. uncovers the complex mechanisms behind this association, revealing how an excess of cholesterol can trigger periodontal inflammation and alveolar bone loss. The researchers found that cholesterol dysregulation in periodontal cells, caused by increased CXCL16-mediated cholesterol influx and production of oxysterol metabolites by CH25H, stimulates the release of inflammatory mediators. This can lead to systemic diseases when released into the bloodstream. The study also highlighted the role of nuclear receptor RORα in mediating periodontitis pathogenesis triggered by cholesterol and its metabolites. These findings pave the way for new therapeutic approaches targeting individual molecular pathogenesis pathways in periodontitis treatment. This summary was initially drafted using artificial intelligence, then revised and fact-checked by the author.

## Introduction

Periodontitis (PD), a type of periodontal disease, is a chronic inflammatory disorder of the gingiva and supporting tissues of the teeth due to specific pathogenic bacteria^1^. It is characterized by inflammatory responses, progressive periodontal destruction, and alveolar bone loss (ABL)^1^. It is a multifactorial disease caused by the interplay between bacterial infection and host immune responses^1,2^. Infection with periodontal bacteria and their metabolites triggers an increased expression of the inflammatory mediator prostaglandin E_2_ (PGE_2_), in addition to pro-inflammatory cytokines, such as interleukin (IL)1β, IL6, and tumor necrosis factor (TNF)α^3,4^, and chemokines such as IL8 and C-C motif chemokine ligand 5 (CCL5)^5^. Matrix metalloproteinases (MMPs), including MMP1 and MMP3, are mainly responsible for periodontal tissue destruction^6,7^. The prevalence of PD depends on individual genetic susceptibility, environmental factors, and systemic disorders, including age, gender, pregnancy, stress, smoking, obesity, diabetes, and cardiovascular and other metabolic diseases^8,9^.

Studies have focused on the bidirectional relationship between periodontal diseases and metabolic syndromes, including diabetes, hypertension, hyperlipidemia, and visceral obesity^10^. PD initiates systemic inflammation. Specifically, the periodontal infection-mediated release of pro-inflammatory cytokines into the systemic circulation^11,12^ alters blood lipid levels and induces abnormal lipid metabolism^13^; consequently, it causes systemic disorders, such as cardiovascular disease, atherosclerosis, and diabetes mellitus^14,15^. Conversely, hyperlipidemia results in disorders via immune dysregulation and oxidative stress, which potentially increase susceptibility to PD^16,17^. It is one of the major risk factors for these diseases^18–21^, and earlier meta-analyses and meta-regression studies on patients revealed a relationship between PD and dyslipidaemia^22^. Consistently, increased acinar inflammation in the liver and hyperlipidemia have been observed in a lipopolysaccharide-induced rabbit model of PD, and a high-fat diet exacerbates ABL, which also supports the bidirectional relationship between PD and hyperlipidemia^23^.

In various cellular processes, cholesterol functions as a structural component of the cell membrane and serves as a precursor molecule for the biosynthesis of bile acids, steroid hormones, and fat-soluble vitamins^24^. Given the range of functions of cholesterol in normal physiology, the disruption of cholesterol homeostasis, particularly hypercholesterolemia, has emerged as a considerable factor contributing to various human diseases. Recently, our group demonstrated that the disruption of cholesterol metabolism is one of the causes of the pathogenesis of osteoarthritis, a well-known degenerative disease^25^. Cellular cholesterol homeostasis is tightly regulated by its synthesis, influx, efflux, and metabolism^24^. In cholesterol metabolism, several types of cholesterol hydroxylases convert cellular cholesterol to oxysterols, which regulate diverse biological processes^24,26^. Previous studies suggested the physiological functions of oxysterols as signaling molecules that maintain the balance of lipid levels in cells and the regulation of cell fate^27^. Furthermore, oxysterols contribute to the pathogenesis of numerous disease processes, including atherosclerosis, neurodegenerative diseases, inflammatory bowel diseases, and degenerative retinal changes^28^. However, the potential mechanisms directly linking cholesterol or its metabolites with PD pathogenesis remain unknown.

PD as a manifestation of a systemic disease is one of the categories of PD defined by the American Academy of Periodontology 1999 classification system^29^. In this study, the direct association between hypercholesterolemia and periodontal inflammation was established on the basis of evidence supporting the dysregulation of cholesterol homeostasis and metabolic alterations during PD pathogenesis.

## Materials and methods

### Human subjects

Human gingival tissue samples comprising both epithelial and connective tissue were collected from twelve subjects (34–65 years old; 49.17 ± 12.40 years), including six healthy individuals for non-inflamed gingival tissue and six patients with chronic periodontitis for inflamed gingival tissue. The research was accepted by the Institutional Review Board of Chonnam National University Dental Hospital (Gwangju, Republic of Korea, #DTMP-2022-005). Patients were explained fully about the procedures and confirmed in the consent. The tissue of gingiva was promptly preserved in liquid nitrogen and stocked at ‑80 °C until the experiments were performed.

### Mice

C57BL/6 male and female mice were fed a high-cholesterol diet with or without cholesterol-lowering drugs. Homozygous Ch25h knockout (*Ch25h*^−/−^) mice^30^ were purchased from the Jackson Laboratory. Male C57BL/6 (wild-type and *Ch25h*^−/−^) mice were specifically used for the induction of experimental periodontitis. Male mice (five weeks old) were fed for 13 weeks with an AIN-76A diet (Feed Laboratory) as a regular diet. For the high-cholesterol diet, mice were fed for 13 weeks with a modified AIN-76A diet supplemented with 2% cholesterol. A group of mice fed the 2% cholesterol diet were injected intragingivally with 15 mg kg^−1^ DMSO (Sigma‒Aldrich, D2650) according to body weight as a vehicle or SR3335 (Cayman Chemical, 12072), a selective RORα inverse agonist, once per week for 13 weeks. All animals were housed in pathogen-free barrier facilities. The study protocols were approved by the Animal Care and Ethics Committee of Chonnam National University.

### Experimental periodontitis (PD) induction in mice

An experimental PD model using male mice (10 weeks old) was established via silk ligature 5-0 (Ailee, SK521). The ligature encircled the right second molar in the maxilla, and the left side was used as the control^31^. Experimental PD was also induced via intragingival injection (once per day for 6 days) of adenoviruses Ad-*Ch25h* and Ad-*Rorα* (1 × 10^9^ PFU in a total volume of 5 μl and 8 µl, respectively) between the first and the second molar of 12-week-old male mice using a Hamilton syringe (32 GA, 9.25 mm, 20°). The control side was injected with empty adenovirus (Ad-C). A group of mice with ligature-induced PD were injected intragingivally with 15 mg kg^−1^ according to body weight of SR3335 once per day for 7 days. The mice were sacrificed, and the maxilla was harvested for μCT and histological analyses after 9 days of ligature^31^. All adenoviral vectors were supplied by Vector Biolabs.

### Primary cell culture

Primary human gingival fibroblasts (GFs) were collected from the marginal gingival tissue of clinically healthy individuals. Participants were notified about the purpose of this study, which was performed by the Institutional Review Board of Chonnam National University Hospital (#DTMP-2022-005). Gingival tissue was minced mechanically using a scalpel in phosphate-buffered saline solution (PBS) and instantly cultured in Dulbecco’s modified Eagle’s medium (DMEM, Gibco, 12800-017) with 10% fetal bovine serum (FBS, Capricon; containing approximately <50 mg/dl cholesterol and 3.0 mg/dl LDL), 1% penicillin and 1% streptomycin. Human GFs were nurtured at 37 °C in a humidified atmosphere of 5% (v/v) CO_2_ and used for experiments at a maximum passage of 9. Cells were treated with cholesterol (cholesterol–methyl-β-cyclodextrin, Sigma‒Aldrich, C-4951) for 24 h or 36 h, 25-hydroxycholesterol (25-HC, Sigma‒Aldrich, H1015), *Pg* lipopolysaccharides (LPS) (InvivoGen, tlrl-pglps) and *Ec* LPS (Sigma‒Aldrich, L2630), IL1β (GenScript, 14 Z02922−10) or TNFα (GenScript, Z02774-20) for 24 h. Human GFs were infected with *CH25H-*, *CYP7B1-*, and *RORα*-overexpressing or control adenovirus (Ad-C) for 2 h, followed by culture for 48 h. Control or *CH25H* siRNA (Dharmacon RNA Technologies, L-008288-01) was transfected into human GFs seeded in six-well plates (2.5 × 10^5^ cells per well) using Lipofectamine™ RNAiMAX (Invitrogen, 56532) according to the manufacturer’s protocol, in the presence of IL1β (2 ng ml^−1^) or TNFα (50 ng ml^−1^). Human GFs were treated with a cholesterol synthesis inhibitor [triparanol 1 μM or 5 μM (Cayman, 20918) or lovastatin 1 μM or 10 μM (Enzo, BML-G245)] or anti-CXCL16 blocking antibody 1 μg ml^−1^ or 2.5 μg ml^−1^ (R&D Systems, AF976) in the presence of IL1β (2 ng ml^−1^) or TNFα (50 ng ml^−1^).

### Microcomputed tomography (μCT)

The maxillae were collected and fixed in 10% neutral buffered formalin (NBF) overnight. The structure of alveolar bone was evaluated by using the SkyScan 1172 μCT scanner (SkyScan) with the following parameters: 49 kV, 200 μA, 0.7 mm aluminum filter, and 11 μm resolution. μCT data were analyzed between the first and second molar regions on the buccal and lingual sides after drawing the region of interest (ROI) using CTAn (SkyScan) and Mimics 14.0 (Materialise).

### Histology and immunohistochemistry

Gingival tissues from patients and mice with or without PD were fixed in 10% NBF for 24 h, embedded in paraffin, and sectioned at 5 μm thickness. Mouse maxillary tissues were decalcified with 0.5 M ethylenediamine tetra-acetic acid (EDTA, pH 8.0) for 2 weeks, embedded in paraffin, and sectioned at 5 μm thickness. Alveolar bone erosion was evaluated via hematoxylin and eosin (H&E) staining. For immunohistochemical staining, endogenous peroxidases were quenched in sections of human and mouse periodontal tissues, and epitope retrieval was induced by 3% H_2_O_2_ for 10 min and 0.1% trypsin for 30 min at 37 °C. The sections were incubated with mouse anti-MMP1 (Novus, NBP2-22123; 1:150), rabbit anti-IL6 (Novus, NB600−1131; 1:150), rabbit anti-IL8 (Biorbyt, orb229133; 1:150), rabbit anti-PTGS2 (Cayman, 160106; 1:150), rabbit anti-RORα (Sigma‒Aldrich, AV45608; 1:150), rabbit anti-CH25H (Bioss, bs-6480R; 1:150), and rabbit anti-CXCL16 (Genetex, GTX116706; 1:150), followed by staining with EnVision+System-HRP and AEC+ substrate (Dako, K4005, K4009) and counterstaining with hematoxylin (Dako, S3309). Images were captured by using a Zeiss Axio Scope A1 microscope and quantified by ImageJ software (National Institutes of Health, v.1.51a).

### Cholesterol quantification and lipid profiling

For determination of cellular cholesterol levels, human GFs were treated with IL1β, TNFα, cholesterol synthesis inhibitors (triparanol (Cayman) and lovastatin (Enzo), or anti-CXCL16 blocking antibody (R&D Systems) in the presence of IL1β (2 ng ml^−1^), TNFα (50 ng ml^−1^) or cholesterol (200 μM). The quantity of intracellular cholesterol was determined by a cholesterol quantification kit (Biomax, BM-CHO−100). Briefly, cellular lipids were extracted with a mixture of chloroform, isopropanol, and NP-40 (7:11:0.1) in a microhomogenizer and centrifuged for 10 min at 15,000 × *g*. The organic phase was collected and dried under vacuum at 55 °C for 12 h. From the lipid extract obtained, the amounts of total and free cholesterol were determined using a total cholesterol quantification kit following the manufacturer’s instructions (Biomax). The cholesteryl ester content was calculated as the total cholesterol minus free cholesterol.

### Cholesterol and lipoprotein imaging

The influx of cholesterol or oxidized low-density lipoprotein (OxLDL) into the cell was evaluated via microscopy. Briefly, human GFs were seeded on a poly-L-lysine-coated culture cover glass (Paul Marienfeld GmbH & Co. KG, 0111520) and stimulated with 5 μg ml^−1^ 1,1-dioctadecyl-3,3,3′,3′-tetramethylindocarbocyanine perchlorate oxidized low-density lipoprotein (Dil-OxLDL, Kalen Biomedical, 770232-9) for 24 h. After washing three times with PBS and fixing with 4% ice-cold fresh paraformaldehyde (PFA, Sigma‒Aldrich, P6148), cell nuclei were stained with DAPI (l μg ml^−1^) for 10 min. For cholesterol staining in periodontal tissue, frozen sections of gingival tissues from human patients and experimental PD mice (10 μm) were washed in PBS and fixed in 4% fresh PFA. Gingival tissue sections were stained with filipin (250 mg ml^−1^; Sigma‒Aldrich, F9765) and washed twice with PBS. All procedures were performed in the dark and at room temperature. Human GFs and periodontal tissues were examined by using a Zeiss Axio Scope A1 microscope attached to the fluorescence part, and the acquired images were analyzed using ImageJ software.

### Enzyme-linked immunosorbent assay (ELISA)

To quantify the secretion of MMP1, IL6, IL8 and the production of PGE_2_ from human GFs, ELISA kits for MMP1 (RayBiotech, #ELH-MMP1−1), IL6 (R&D Systems, D6050), IL8 (R&D Systems, D8000C) and PGE_2_ (R&D Systems, KGE004B) were used. Briefly, human GFs were cultured without serum under each experimental set of conditions. The culture medium was collected and centrifuged for 10 min at 1200 × *g*, and the supernatant fractions were stored at ‑80 °C until analysis. The MMP1, IL6, IL8, and PGE_2_ levels were determined following the manufacturer’s instructions.

### RNA isolation and real-time quantitative polymerase chain reaction (RT‒qPCR)

Total RNA from primary cultures of human GFs and gingiva was isolated using TRIzol reagent (Sigma‒Aldrich, 93289). The synthesis of cDNA was performed from 0.25 to 0.5 μg of RNA via reverse transcription (Promega, A3803) and subjected to PCR (Geneall, 501-025). The Supplementary Table lists the PCR primers and experimental conditions. Quantitative RT‒PCR was executed with SYBR Premix Ex Tag (Takara Bio, RR420A) using a StepOnePlus Real-Time PCR system (Thermo Fisher Scientific). All qRT-PCR reactions were performed in duplicate, and fold changes were calculated using relative quantification methods with glyceraldehyde-3-phosphate dehydrogenase (GAPDH) serving as an internal control.

### Western blotting

Human GFs were lysed in RIPA buffer, a protease inhibitor cocktail (Roche, 04906845001), and a phosphatase inhibitor cocktail (Roche, 11697498001). Briefly, 30 µg of protein was separated by SDS‒PAGE using 8%, 10%, and 15% PAGE gels, and the blots were incubated with the indicated antibodies. As a loading control, mouse anti-β-actin (Sigma‒Aldrich, A3854) was used. The membranes were incubated with the following antibodies: rabbit anti-MMP1 (Abnova, PAB12708), rabbit anti-IL6 (Novus), rabbit anti-IL8 (Biorbyt), rabbit anti-PTGS2 (Cayman), rabbit anti-RORα (Santa Cruz, Sc-28612), and rabbit anti-CH25H (Bioss). After washing with Tris-buffered saline with 0.1% Tween (TBS-T), the membranes were incubated with horseradish peroxidase-conjugated secondary antibodies (anti-rabbit IgG; Sigma‒Aldrich, A6154) and detected using an ECL solution (Cytiva, RPN2235). Images were obtained using ImageSaver6 software via an EZ capture MG system (ATTO) and were quantified using ImageJ software.

### Reporter gene assay

The RORE motif (50 ng per well, ActiveMotif, 32175) was transfected into human GFs using Lipofectamine^TM^ RNAiMAX (Invitrogen). After recovery from transfection, human GFs were stimulated with IL1β (1 ng ml^−1^), TNFα (25 ng ml^−1^), cholesterol (100 μM), and 25-HC (20 μM) or were infected with 800 MOI of Ad-C or Ad-*CH25H* for 36 h. Renilla luciferase activities were measured by the LightSwitch^TM^ Luciferase assay kit (ActiveMotif, 32031).

### Microarray analysis

Total RNA was extracted from human GFs treated with IL1β (2 ng ml^−1^) or TNFα (50 ng ml^−1^), and the concentration, purity, and integrity were verified by spectrophotometry. Three replicates for each experimental group were isolated and processed. RNA from human GFs was analyzed using the Affymetrix GeneChip Mouse Gene 2.0 ST Array following the Affymetrix protocol (Macrogen). Data were normalized using algorithms supplied with the feature extraction software. The cutoff values for the identification of differentially expressed genes were an adjusted *P* value below 0.05 (FDR < 0.05) and an absolute value of fold change over 1.5 (|FC|>1.5).

### Statistical analysis

All experiments were repeated at least three times. Values are presented as the mean ± SEM. Statistical analyses were analyzed by GraphPad Prism version 8 (GraphPad Software, Inc.). Quantified data were initially tested for conformation to a normal distribution using the Kolmogorov‒Smirnov or Shapiro‒Wilk test, followed by analysis with two-tailed Student’s *t*-test for comparison of means between two groups or analysis of variance (ANOVA) followed by Tukey’s post hoc test (multi-comparison) for comparison of means among three or more independent groups, as appropriate. The *n* value represents the number of independent experiments or mice. Differences with *P* values < 0.05 were assigned statistically significant.

## Results

### Cholesterol is a potential cause of PD

**Fig. 1:**
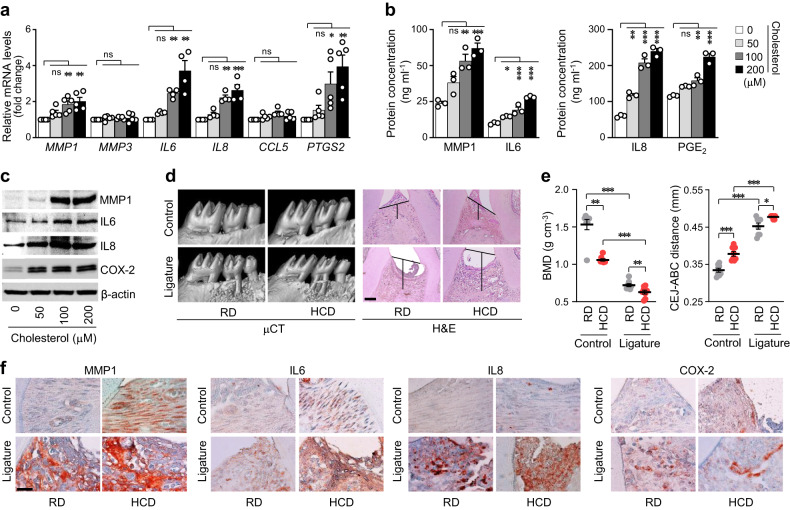
High cholesterol levels cause periodontal inflammation and alveolar bone loss. mRNA (**a**) and protein (**b**, **c**) levels of the indicated molecules in human GFs treated with cholesterol for 36 h (*n* ≥ 3). **d**, **e** Representative μCT images showing the results of BMD analysis and H&E staining images presenting the CEJ-ABC distance measurements in the maxilla region of ligature-induced PD mice fed a regular diet (RD) or high-cholesterol diet (HCD) for 13 weeks (*n* = 8). Scale bar, 100 μm. **f** Representative images of MMP1, IL6, IL8, and COX-2 immunostaining from ligature-induced PD mice fed an RD or HCD. Magnification, ×1000. Scale bar, 25 μm. *n* indicates the number of biologically independent samples and mice per group. Values are presented as the mean ± SEM via two-tailed *t*-test (**e**) and one-way ANOVA with Tukey’s test (**a**, **b**). (*P < 0.05, **P < 0.01, ***P < 0.001).

In this study, the pathogenic mechanisms of PD involving abnormal cholesterol metabolism were investigated to explore the association between PD pathogenesis and dyslipidemia. Since PD accompanies gingival inflammation, the expression levels of pro-inflammatory factors (*MMP1*, *MMP3*, *IL6*, *IL8*, *CCL5*, and *PTGS2*) were analyzed following exogenous cholesterol treatment of human GFs. GFs are among the most predominant cell types in periodontal tissues and are essential for maintaining the infection status^32^. In the present study, when primary cultures of human GFs were directly treated with cholesterol, the expression and activity of pro-inflammatory factors associated with PD were significantly increased (Fig. 1a–c, Supplementary Fig. 1a). PD is characterized by the following major phenotypes: a decrease in bone mineral density (BMD) and an increase in ABL, which is expressed as the cementum-enamel junction to the alveolar bone crest (CEJ-ABC) distance measured via μCT or histological analysis^33^. In vivo experiments were performed on a ligature-induced PD mouse model to determine the effects of high cholesterol feeding on BMD and ABL. High cholesterol diet (HCD)-fed mice showed BMD loss and increased ABL (Fig. 1d, e) compared with those of regular diet (RD)-fed mice in the control group. Moreover, the HCD-fed mice exhibited marked inflammation and bone loss in the ligature-induced PD groups (Fig. 1d, e). Experimental PD phenotypes were further validated on the basis of the upregulation of MMP1, IL6, IL8, and COX-2 in gingival tissues, as assessed via immunohistochemistry. The expression of these inflammatory proteins was higher in the HCD groups of the control and PD models (Fig. 1f, Supplementary Fig. 1b). Therefore, the collective data suggests that excess cholesterol is a potential cause of periodontal inflammation and alveolar bone erosion.

### Cellular cholesterol is increased by uptake via CXCL16 in gingival tissues

**Fig. 2:**
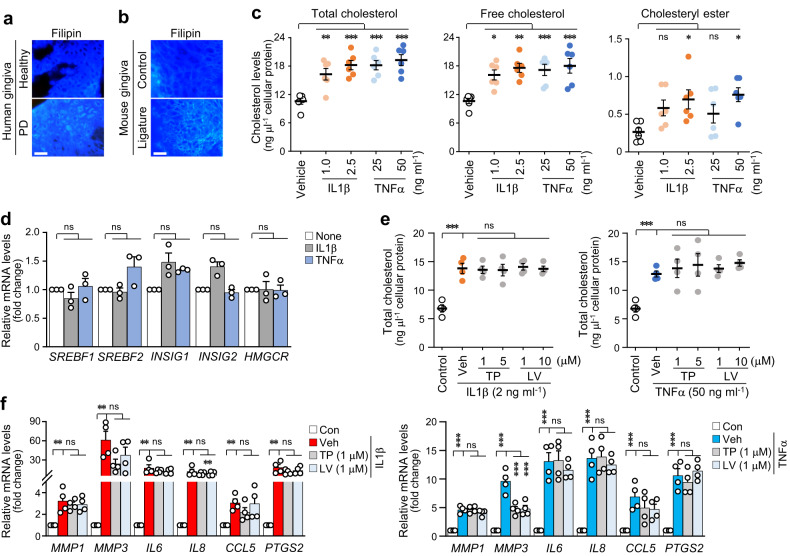
PD-associated catabolic signaling increases cholesterol uptake into periodontal cells. Representative images of filipin staining of gingival tissue from human patients with PD (**a**) and in the maxilla region of ligature-induced PD mice (**b**). Scale bar, 25 μm. Total, free cholesterol, and cholesteryl ester (*n* = 6) levels (**c**) and mRNA (*n* = 3) (**d**) levels of cholesterol synthesis-related genes in human GFs treated with IL1β or TNFα. **e** Total cholesterol level in human GFs treated with the cholesterol synthesis inhibitors triparanol (TP) or lovastatin (LV) in the presence of IL1β (2 ng ml^−1^) or TNFα (50 ng ml^−1^) for 24 h (*n* = 4). **f** mRNA levels of *MMP1*, *MMP3*, *IL6*, *IL8*, *CCL5*, and *PTGS2* in human GFs treated with cholesterol synthesis inhibitors (TP and LV) in the presence of IL1β (2 ng ml^−1^) or TNFα (50 ng ml^−1^) for 24 h (*n* = 4). *n* indicates the number of biologically independent samples. Values are presented as the mean ± SEM based on one-way ANOVA with Tukey’s test. (*P < 0.05, **P < 0.01, ***P < 0.001).

**Fig. 3:**
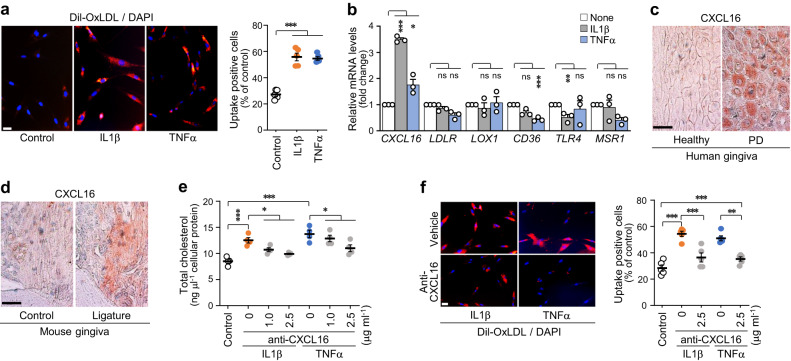
Upregulation of CXCL16 in inflamed periodontal cells and tissues and the CXCL16-mediated increase in cellular cholesterol in GFs. **a** Representative images and measurement of Dil-OxLDL uptake into human GFs treated with or without IL1β (2 ng ml^−1^) or TNFα (50 ng ml^−1^) for 24 h (*n* = 5). Scale bar, 25 μm. **b** Transcript levels of cholesterol uptake receptors in human GFs treated with IL1β (5 ng ml^−1^) or TNFα (100 ng ml^−1^) (*n* = 3). Representative images of CXCL16 immunostaining of the gingiva from human patients with PD (**c**) and ligature-induced PD mice (**d**). Total cholesterol levels (*n* = 4) (**e**) and measurement of Dil-OxLDL uptake into human GFs (*n* = 5) (**f**) treated with an anti-CXCL16 blocking antibody in the presence of IL1β (2 ng ml^−1^) or TNFα (50 ng ml^−1^) for 24 h. *n* indicates the number of biologically independent samples. Values are presented as the mean ± SEM based on a two-tailed *t*-test (**a**) and one-way ANOVA with Tukey’s test (**b**, **e**, **f**). (*P < 0.05, **P < 0.01, ***P < 0.001).

The events associated with cholesterol homeostasis were investigated to explore the mechanism by which hyperlipidemia regulates the PD phenotype. Staining with a cholesterol-specific dye (filipin) revealed an increase in cellular deposits of cholesterol in the inflamed gingiva compared with that in healthy tissues of humans (Fig. 2a). This finding was supported by the results obtained with the PD mouse model (Fig. 2b). PD-mimicking conditions were induced in human GFs through treatment with the pivotal virulence factor LPS or representative pro-inflammatory cytokines, mainly IL1β or TNFα. The extent of inflammation and alveolar bone loss in LPS-induced periodontal disease is primarily influenced by the upregulation of pro-inflammatory cytokines. Intracellular cholesterol levels, including total cholesterol and free cholesterol, were increased by treatment with LPS (*Pg* LPS and *Ec* LPS) (Supplementary Fig. 2a) and pro-inflammatory cytokines (IL1β and TNFα) in human GFs (Fig. 2c). This increase in intracellular cholesterol could be explained by an increase in cholesterol synthesis or increased cholesterol influx. Microarray analysis was performed on human GFs stimulated with IL1β or TNFα (data not shown) to determine the expression patterns of genes associated with cholesterol metabolism. The expression levels of cholesterol synthesis-related genes, such as *SREBF1*, *SREBF2*, *INSIG1*, *INSIG2*, and *HMGCR*, were not significantly different between the cytokine-stimulated and control cell groups (Fig. 2d, Supplementary Fig. 2b, c). Moreover, the increased cellular cholesterol levels (Fig. 2e, Supplementary Fig. 2d, e) and the expression of pro-inflammatory factors (Fig. 2f) induced by IL1β or TNFα were not altered after cholesterol biosynthesis in human GFs was inhibited by triparanol and lovastatin. Interestingly, trafficking analysis using exogenous fluorescence-labeled oxidized LDL (Dil-OxLDL; Fig. 3a) demonstrated that cellular cholesterol levels in IL1β- and TNFα-stimulated human GFs were markedly elevated because of increased cholesterol uptake. Accordingly, we examined the expression patterns of differentially expressed genes (DEGs) associated with cholesterol influx from the microarray data (data not shown). Among the studied cholesterol uptake-related genes, C-X-C motif chemokine ligand (CXCL) 16, a receptor of OxLDL that contains cholesterol^34^, was significantly upregulated in response to PD-associated signaling in human GFs (Fig. 3b, Supplementary Fig. 3a, b). As a scavenger receptor showing upregulation in periodontal tissues, CXCL16 was further examined in the inflamed human gingiva and the experimental mouse PD model (Fig. 3c, d, Supplementary Fig. 3c, d). CXCL16 was neutralized with a specific blocking antibody, thereby validating its function in exogenous cholesterol influx. The cytokine-stimulated increase in intracellular cholesterol was blocked upon treatment with the CXCL16-specific antibody in inflamed human GFs (Fig. 3e, Supplementary Fig. 3e). This observation was further supported by the finding that the Dil-OxLDL influx was inhibited after antibody treatment (Fig. 3f). Therefore, these data suggest that the CXCL16-mediated uptake of OxLDL contributes to the increased level of cellular cholesterol in gingival tissues with periodontal inflammation.

### Increased production of oxysterol metabolites contributes to PD pathogenesis

**Fig. 4:**
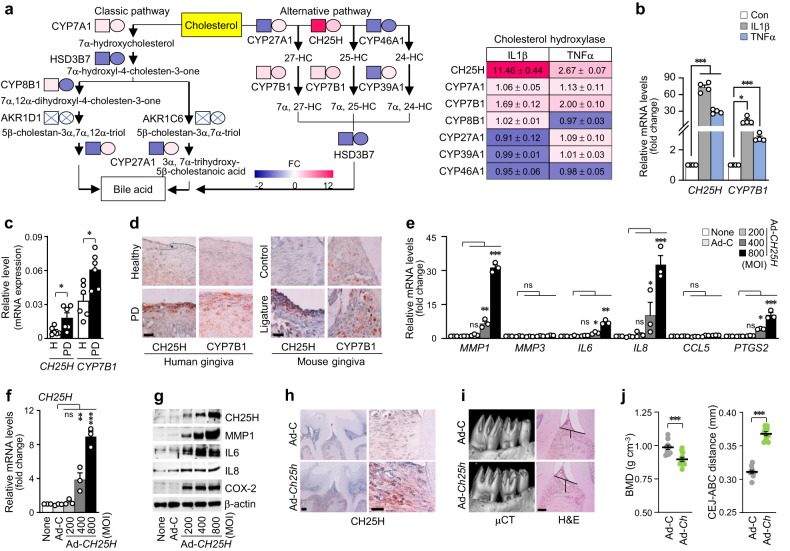
CH25H upregulation is implicated in PD pathogenesis. **a** Heatmaps of genes involved in cholesterol metabolism and metabolic pathways of bile acid synthesis from the microarray analysis of human GFs treated with IL1β (left square) and TNFα (right circle) (*n* = 3). Transcript levels of *CH25H* and *CYP7B1* in human GFs treated with IL1β (5 ng ml^−1^) and TNFα (100 ng ml^−1^) for 24 h (*n* = 4) (**b**) and the gingiva of healthy patients and patients with PD (*n* = 6) (**c**). **d** Representative immunostaining images of CH25H and CYP7B1 in human and mouse gingiva. Scale bar, 25 μm. **e**, **f** mRNA levels in human GFs infected with empty (Ad-C) or *CH25H*-encoding adenovirus (Ad-*CH25H*) (*n* = 3) for 48 h (*n* = 3). **g** Protein levels of CH25H, MMP1, IL6, IL8, and PTGS2 in cell lysates of human GFs infected with *CH25H*-overexpressing adenovirus (Ad-*CH25H*; *n* = 3). **h**, **i** Representative images of CH25H immunostaining, μCT, and H&E staining images of mouse gingiva injected with an empty virus (Ad-C) or Ad-*Ch25h* (1 × 10^9^ PFU per 5 μl). Magnification, ×100. Scale bar, 100 μm (left). Scale bar, 25 μm (right) (**h**). Magnification, ×200. Scale bar, 100 μm (**i**). **j** BMD analysis and CEJ-ABC distance measurement of the mouse maxillae injected intragingivally with Ad-C and Ad-*Ch25h* (1 × 10^9^ PFU per 5 μl; *n* = 10). Scale bar, 100 μm. *n* indicates the number of biologically independent samples and mice per group. Values are presented as the mean ± SEM based on a two-tailed *t*-test (**c**, **j**) and one-way ANOVA with Tukey’s test (**b**, **e**, **f**). (*P < 0.05, **P < 0.01, ***P < 0.001).

**Fig. 5:**
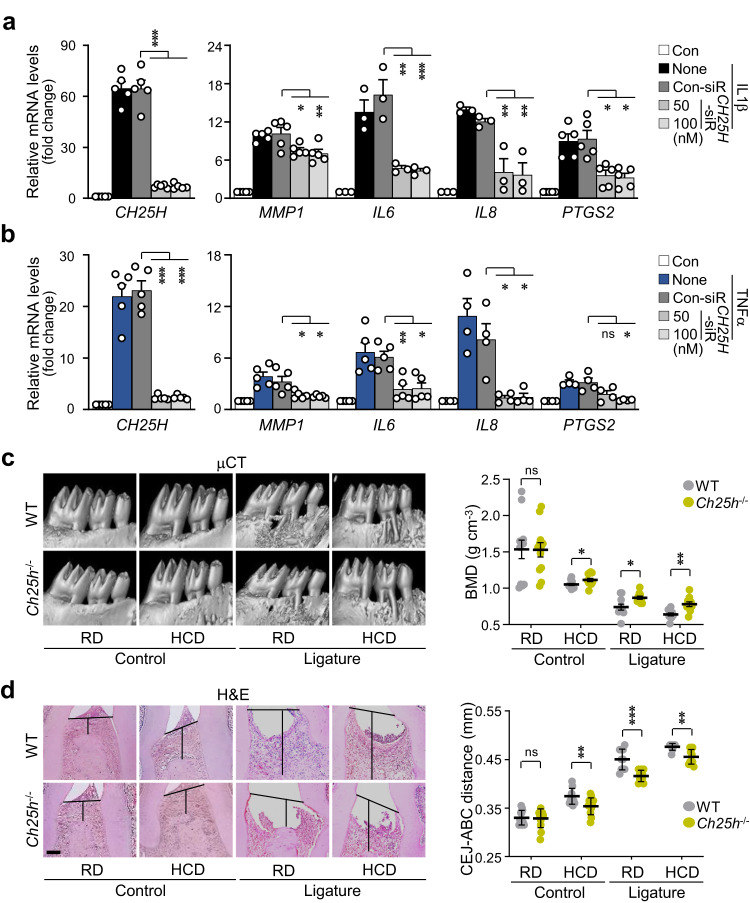
Blocking CH25H alleviates periodontal inflammation and bone loss. mRNA levels of *CH25H*, *MMP1*, *IL6*, *IL8*, and *PTGS2* in human GFs transfected with *CH25H* siRNA in the presence of IL1β (2 ng ml^−1^) (**a**) or TNFα (50 ng ml^−1^) (**b**) for 24 h (*n* ≥ 3). Representative μCT (**c**) and H&E staining images (**d**) for BMD analysis and CEJ-ABC distance measurement of the maxillae of ligature-induced PD wild-type (WT) and *CH25H* knockout (*Ch25h*^−/−^) mice (*n* ≥ 10). Scale bar, 100 μm. *n* indicates the number of biologically independent samples and mice per group. Values are presented as the mean ± SEM based on a two-tailed *t*-test (**c**, **d**) and one-way ANOVA with Tukey’s test (**a**, **b**). (*P < 0.05, **P < 0.01, ***P < 0.001).

Oxysterols, which are oxygenated cholesterol derivatives, participate in various inflammatory diseases, including neuro-inflammatory disorders, atherosclerosis, and obesity^26^. We investigated the potential association between cholesterol metabolism and PD by comparing alterations in the gene expression patterns of various isotypes of cholesterol hydroxylases. On the basis of microarray analysis, we generated a heatmap of cholesterol hydroxylases that function in the metabolic pathways of bile acid synthesis in the liver (Fig. 4a). Among the genes examined, those encoding the cholesterol hydroxylases *CH25H* and *CYP7B1* were the most significantly upregulated by IL1β (left square) and TNFα (right circle) in human GFs (Fig. 4a). We further confirmed the upregulation of *CH25H* and *CYP7B1* via qRT‒PCR analysis (Fig. 4b, Supplementary Fig. 4a, b) and examined them in the inflamed gingiva of patients with PD and ligature-induced mouse PD models (Fig. 4c, d, Supplementary Fig. 4c, d). Next, we explored the effects of the gain-of-function of CH25H and CYP7B1 on inflammatory gene expression and PD pathogenesis. The expression levels of *MMP1*, *IL6*, *IL8*, and *PTGS2* increased upon CH25H overexpression via adenovirus infection in human GFs (Fig. 4e–g, Supplementary Fig. 4e). However, unexpectedly, infection with adenovirus encoding *CYP7B1* did not induce significant changes in pro-inflammatory factor expression (Supplementary Fig. 4f). CH25H catalyzes the conversion of cholesterol to 25-hydroxycholesterol (25-HC). The data obtained from the treatment with 25-HC confirmed the effects of CH25H overexpression (Supplementary Fig. 4g). Intragingival injection of Ad-*Ch25h* (Fig. 4h, Supplementary Fig. 4h) led to the development of PD phenotypes characterized by increased BMD loss and ABL, as evidenced by the increased CEJ-ABC distance (Fig. 4i, j). *CH25H* knockdown with siRNA alleviated IL1β- and TNFα-stimulated inflammatory gene expression (Fig. 5a, b). We further elucidated the in vivo catabolic role of CH25H in PD pathogenesis by using *Ch25h* knockout (KO) mice (*Ch25h*^*−/−*^). The manifestations of ligature-induced PD, such as periodontal inflammation and subsequent alveolar bone erosion, were significantly reduced in *Ch25h*^*−/−*^ mice compared with those of their WT littermates (Fig. 5c, d). Immunohistochemistry revealed that inflammatory genes and pro-inflammatory factors, such as MMP1, IL6, IL8, and COX-2, were also upregulated in gingival tissues (Supplementary Fig. 5). Moreover, PD pathogenesis was further promoted by HCD feeding for 13 weeks in WT mice, but it was alleviated in *Ch25h*^*−/−*^ mice (Fig. 5c, d, Supplementary Fig. 5). Our data collectively suggest that the increased oxysterol metabolite production by CH25H contributes to PD pathogenesis.

### RORα mediates cholesterol-induced PD pathogenesis

**Fig. 6:**
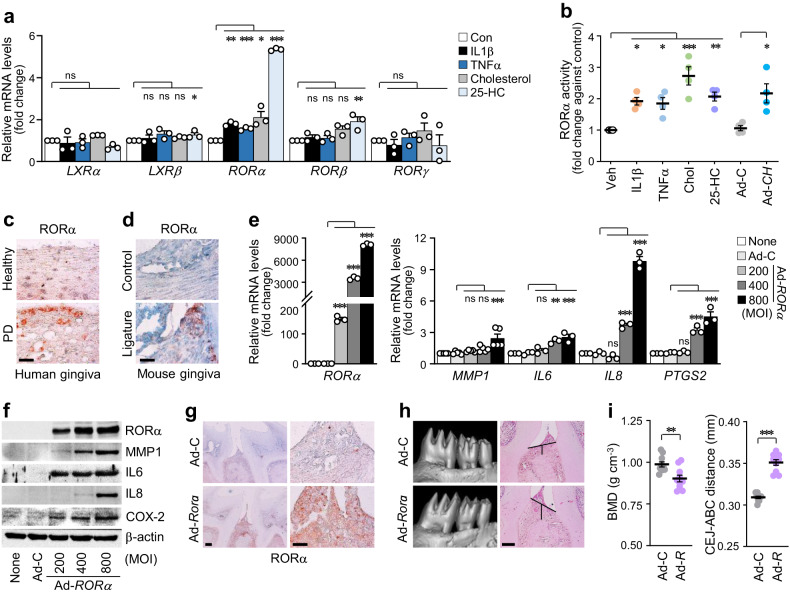
RORα mediates cholesterol-induced PD pathogenesis. **a** Transcript levels of oxysterol-binding nuclear receptors in human GFs treated with IL1β (5 ng ml^−1^), TNFα (100 ng ml^−1^), cholesterol (200 μM), or 25-HC (20 μM) for 24 h (*n* = 3). **b** RORα transcriptional activity in human GFs treated with IL1β (2 ng ml^−1^), TNFα (50 ng ml^−1^), cholesterol (100 μM), and 25-HC (20 μM) for 24 h or infected with Ad-*CH25H* (800 MOI; *n* = 4). Representative RORα immunostaining images of the gingiva of human patients with PD (**c**) and ligature-induced PD mice (**d**). Scale bar, 25 μm. mRNA (**e**) and protein (**f**) levels in human GFs infected with Ad-*RORα* for 48 h (*n* ≥ 3). Representative RORα immunostaining (**g**), μCT, and H&E staining images (**h**) in mouse gingiva injected with an empty virus (Ad-C) or Ad-*Rorα* (1 × 10^9^ PFU per 8 μl). Scale bar, 100 μm (left). Scale bar, 25 μm (right) (**g**). Scale bar, 100 μm (**h**). **i** Analysis of BMD and the CEJ-ABC distance in mouse maxillae injected intragingivally with Ad-C or Ad-*Rorα* (1 × 10^9^ PFU per 8 μl; *n* = 10). *n* indicates the number of biologically independent samples and mice per group. Values are presented as the mean ± SEM based on a two-tailed *t*-test (**i**) and one-way ANOVA with Tukey’s test (**a**, **b**, **e**). (*P < 0.05, **P < 0.01, ***P < 0.001).

**Fig. 7:**
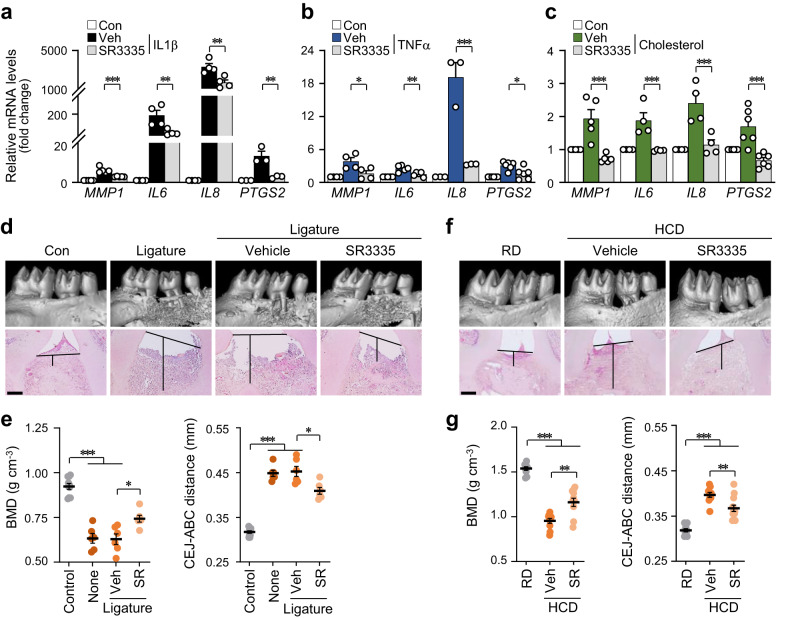
Blockade of RORα activity exerted protective effects against PD phenotypes. mRNA levels of human GFs treated with IL1β (**a**), TNFα (**b**), or cholesterol (**c**) for 24 h (*n* ≥ 3) in the absence or presence of SR3335. Representative μCT and H&E staining images (**d**) of the analysis of BMD and the CEJ-ABC distance (**e**) of the maxillae of ligature-induced PD mice injected intragingivally with DMSO and SR3335 (15 mg kg^−1^; *n* ≥ 6). Scale bar, 100 μm. Representative μCT and H&E staining images (**f**) of the analysis of BMD and the CEJ-ABC distance (**g**) of the maxillae of mice fed a regular diet (RD) or high-cholesterol diet (HCD) and injected intragingivally with DMSO or SR3335 (15 mg kg^−1^; *n* ≥ 10). Scale bar, 100 μm. Values are presented as the mean ± SEM based on a two-tailed *t*-test (**e**, **g**) and one-way ANOVA with Tukey’s test (**a–c**). (*P < 0.05, **P < 0.01, ***P < 0.001).

Given that nuclear receptors respond to excess cellular cholesterol or its oxysterol derivatives through target gene activation^35^, we verified the expression patterns of putative receptors for cholesterol and oxysterols, including the liver X receptor (LXR) and retinoid-related orphan receptor (ROR) families, during PD pathogenesis. Initial screening experiments revealed that *RORα* is specifically increased by PD-associated catabolic signaling, IL1β, TNFα, cholesterol, and 25-HC, thereby prompting further research on the nuclear receptor RORα (Fig. 6a, Supplementary Fig. 6a–d). Previously, we showed that cholesterol or 25-HC binds to RORα and that activated RORα is a direct transcription factor targeting MMPs in another cell type, i.e., chondrocytes, which are specifically found in cartilage^25^. The transcriptional activity of RORα was significantly upregulated by inflammatory cytokines, cholesterol, CH25H, and 25-HC, as determined by the reporter gene assay (Fig. 6b). Consistently, the RORα protein was markedly upregulated in the inflamed gingival tissues of patients with PD and experimental PD mice (Fig. 6c, d, Supplementary Fig. 6e, f). RORα was overexpressed via infection with *RORα*-encoding adenovirus to determine whether RORα regulates the gene expression of pro-inflammatory factors. RORα overexpression increased the mRNA (Fig. 6e) and protein (Fig. 6f, Supplementary Fig. 6g) levels of MMP1, IL6, IL8, and COX-2. Next, the involvement of RORα activation in PD pathogenesis in mice was examined. The data showed a decrease in BMD and an increase in ABL in the mouse model that was intragingivally injected with Ad-*Rorα* (Fig. 6g–i, Supplementary Fig. 6h). The functions of RORα were further explored using SR3335, a selective inverse agonist for RORα^36^. SR3335 treatment substantially reduced the effects of IL1β, TNFα, and cholesterol on the expression of inflammatory mediators (Fig. 7a–c, Supplementary Fig. 7). The effects of RORα activation were confirmed in vivo by intragingival SR3335 injection. The blockade of RORα activity exerted protective effects against ligature-induced PD phenotypes, such as alleviation of the inflammation and ABL (Fig. 7d, e). Additionally, PD phenotypes induced by a HCD, which encompassed increased inflammation and periodontal alveolar bone loss, were also mitigated by inhibiting RORα activity through the use of SR3335 (Fig. 7f, g). Based on these findings, we conclude that RORα mediates PD pathogenesis triggered by cholesterol and its metabolites in mice.

## Discussion

PD is a multifactorial disease with a high prevalence worldwide. PD itself has been considered a local inflammation primarily caused by pathogenic microorganisms in the supporting tissues of teeth; however, the release of inflammatory cytokines by inflamed oral tissues into the blood circulation supports that the potency is associated with other systemic diseases^10,37,38^. Studies on human patients and animal models have suggested an association between PD and various metabolic disorders involved in dyslipidemia, cardiovascular diseases, atherosclerosis, and type II diabetes. Previous reports showed that total cholesterol, LDL-cholesterol, and triglyceride levels are significantly elevated, but HDL-cholesterol levels are reduced in patients with chronic PD; on this basis, recent studies have raised the issue that hyperlipidemia may be a risk factor for PD^22,39^. Although previous meta-analyses of serum cholesterol levels on PD have suggested that PD is positively correlated with dyslipidemia^22^, the obvious cause-and-effect sequence remains unclear. Here, we provided direct evidence that hypercholesterolemia, a type of hyperlipidemia, is a key causative factor of PD development and investigated the underlying mechanisms in detail. Our results indicated that the dysregulation of cellular cholesterol homeostasis is caused by the increased CXCL16-mediated cholesterol influx from the extracellular region, such as blood circulation, and the production of oxysterol metabolites by CH25H, along with RORα activation-mediated transcriptional regulation of pro-inflammatory factors; thus, PD pathogenesis is stimulated.

In the current study, we showed that hypercholesterolemia is a cause of PD pathogenesis. The exogenous treatment of primary cultured human GFs with cholesterol caused PD-like phenotypes and increased the levels of inflammatory mediators, cytokines, and matrix-degrading enzymes, such as MMP1, IL6, IL8, and COX-2, corresponding to the in vivo results obtained with the ligature-induced experimental PD mouse models with or without HCD feeding (Fig. 1). Exposure to HCD for 13 weeks increased BMD loss and ABL in the control group; it further enhanced PD phenotypes in the ligature-induced PD mouse model. These data indicate that abnormal cellular cholesterol accumulation through an increase in extracellular cholesterol or cholesterol influx by pro-inflammatory cytokines stimulates not only PD progression but also susceptibility to PD development.

Cholesterol homeostasis is strictly controlled by the balance of the endogenous synthesis, influx, and efflux of cholesterol. Aside from the de novo synthesis of cholesterol in the liver, dietary cholesterol absorbed from the small intestine is transported to the liver, and hepatocytes secrete lipids in VLDL that are then processed into LDL, the main lipoprotein that delivers lipids to peripheral cells^24^. Cholesterol is obtained from the uptake of circulating plasma LDL via LDL receptor-mediated endocytosis. Free cholesterol is converted to cholesteryl esters by acyl-CoA acyltransferase to maintain cholesterol homeostasis in cells, and cholesteryl esters are stored as cytosolic lipid droplets^40^. In our study, cholesterol was upregulated in inflamed human gingiva and a ligature-induced PD mouse model (Fig. 2a, b); intracellular cholesteryl ester, free cholesterol, and total cholesterol accumulated in human GFs stimulated with IL1β or TNFα, which are well-known pro-inflammatory cytokines increased by LPS from gram-negative bacteria, such as *Porphyromonas gingivalis*^41^. Cellular cholesterol levels in human GFs increased because of the increase in cholesterol influx mediated by CXCL16 upregulation (Fig. 3) rather than endogenous cholesterol synthesis (Fig. 2). Studies have shown that LDL cholesterol is recognized and internalized by several receptors, including CXCL16, LDLR, LOX1, CD36, TLR4, and MSR1, in different cell types^42–44^. We identified the DEGs in the microarray data, used a specific antagonizing antibody, and found that CXCL16 is responsible for the influx of extracellular LDL cholesterol in human GFs (Fig. 3). Cellular cholesterol in inflamed human gingiva, experimental PD mouse gingiva, and IL1β- or TNFα-stimulated human GFs were upregulated because of the increase in the CXCL16 receptor. Verification of CXCL16 function in vivo could provide strong evidence for CXCL16-dependent cholesterol influx during the pathogenesis of periodontal disease. Further experiments to determine the exact role of CXCL16 in PD pathogenesis are warranted. Although the liver is the primary site for cholesterol biosynthesis, a few reports have suggested that endogenous synthesis occurs in peripheral cells^45^. Thus, we investigated the expression of cholesterol biosynthesis-regulating genes, including *SREBF1*, *SREBF2*, *INSIG1*, *INSIG2*, and *HMGCR*, in human GFs stimulated with IL1β or TNFα (Fig. 2). The lack of a change in the biosynthesis of endogenous cholesterol in inflamed human GFs was verified using two specific inhibitors, lovastatin and triparanol. Lovastatin is a type of statin medication used to treat hyperlipidemia by blocking HMG-CoA reductase, which is the rate-limiting enzyme of cholesterol biosynthesis^46^. Triparanol is the first synthetic cholesterol-lowering drug that inhibits the final step in cholesterol biosynthesis^47^. In the present study, these inhibitors did not affect the increased level of cholesterol in inflamed human GFs.

Because there is no direct degradation system for cholesterol in cells, excess cellular cholesterol is removed by efflux and then transported to the liver^24^, or the cellular cholesterol level is decreased by modification, such as hydroxylation^48^. Cholesterol is converted by several types of hydroxylases to oxysterols, which are oxygenated cholesterol derivatives. Oxysterols play markedly diverse roles in biological activities, and the most notable activity is the regulation of cholesterol homeostasis^26^. In our study, CH25H was responsible for cholesterol oxidation in inflamed human GFs, thereby producing 25-HC. To verify the significant role of CH25H and 25-HC in PD pathogenesis in vivo, we generated a ligature-induced PD model with HCD in *Ch25h*^*−/−*^ mice and compared these mice with WT littermates fed a normal diet (Fig. 5, Supplementary Fig. 5). The group of HCD-fed *Ch25h*^*−/−*^ mice presented reduced PD phenotypes, such as BMD loss and ABL, and a decrease in pro-inflammatory factors, such as MMP1, IL6, IL8, and PTGS2, compared with those of the HCD-fed WT littermates. Experimental ligature surgery in the group of *Ch25h*^*−/−*^ mice with HCD exposure induced PD phenotypes; however, the extent of ABL was alleviated compared with that of the group of WT littermates with HCD exposure. Various biological activities of oxysterols appear to be controlled by interactions with different receptors, such as RORs, LXRs, estrogen receptors, and glucocorticoid receptors^49^. On the basis of our microarray data, we found that RORα mainly participates in the 25-HC-mediated expression of catabolic and inflammatory factors. We verified this finding by using SR3335, a selective inverse agonist of RORα.

Our collective findings provide direct evidence of a link between hyperlipidemia and PD pathogenesis. Systemic hypercholesterolemia is a potential causative factor of PD development, and alterations in gene expression induced by inflammatory responses in periodontal cells cooperatively promote PD pathogenesis. These external and internal changes elicit the dysregulation of cholesterol homeostasis in periodontal cells, consequently promoting the release of inflammatory mediators by inflamed periodontal tissues into the blood circulation; potentially, these changes can cause other systemic diseases.

PD and dyslipidemia are affected by age, genetic background, eating habits, nutritional status, and disease status. At present, conventional periodontal therapy comprising surgical and nonsurgical therapy with adjunctive treatment may be ineffective in individuals with various genetic and adaptive backgrounds^50^. Individual molecular pathogenesis pathways should be comprehensively understood, and target-oriented drugs should be developed to establish novel therapeutic approaches for the management of PD. Since metabolic abnormalities, such as hyperlipidemia, alter the oral microbiome and potentiate the deleterious effects of PD, improved knowledge of these associations may markedly expand the scope of oral health and dental practice.

### Supplementary information

Supplementary information
