# Supplementary material for: Disruption of cholesterol homeostasis triggers periodontal inflammation and alveolar bone loss
Source: Exp Mol Med. 2023 Dec 1;55(12):2553–63. doi: 10.1038/s12276-023-01122-w (PMC10767058; doi:10.1038/s12276-023-01122-w)
Supplement: Supplementary file 1 — Supplementary information [file 12276_2023_1122_MOESM1_ESM.pdf]

Supplementary Information

Disruption of cholesterol homeostasis triggers periodontal inflammation and alveolar bone loss

Thanh-Tam Tran[1,2], Gyuseok Lee[1,2], Yun Hyun Huh[3], Ki-Ho Chung[4], Sun Young Lee[1], Ka Hyon Park[1], Seung Hee Kwon[2], Min-Suk Kook[5], Jang-Soo Chun[3], Jeong-Tae Koh[1,2], Je-Hwang Ryu[1,2]*

Supplementary Figure 1 to 7

Supplementary Table

Supplementary Figures

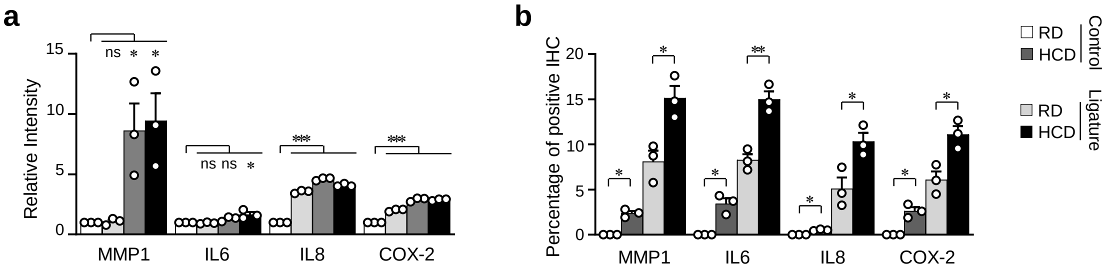

Supplementary Fig. 1. Quantification of western blot and immunohistochemistry staining results. a Relative intensity of the indicated proteins in human GF treated with cholesterol for 36 h (*n* = 3). **b** Percentage of positive staining for MMP1, IL6, IL8, and COX-2 immunostaining in images from ligature-induced PD mice fed with RD or HCD (*n* = 3). Values are presented as mean ± SEM via two-tailed *t*-test (**b**) and one-way ANOVA with Tukey's test (**a**). (**P* < 0.05, ***P* < 0.01, ****P* < 0.001).

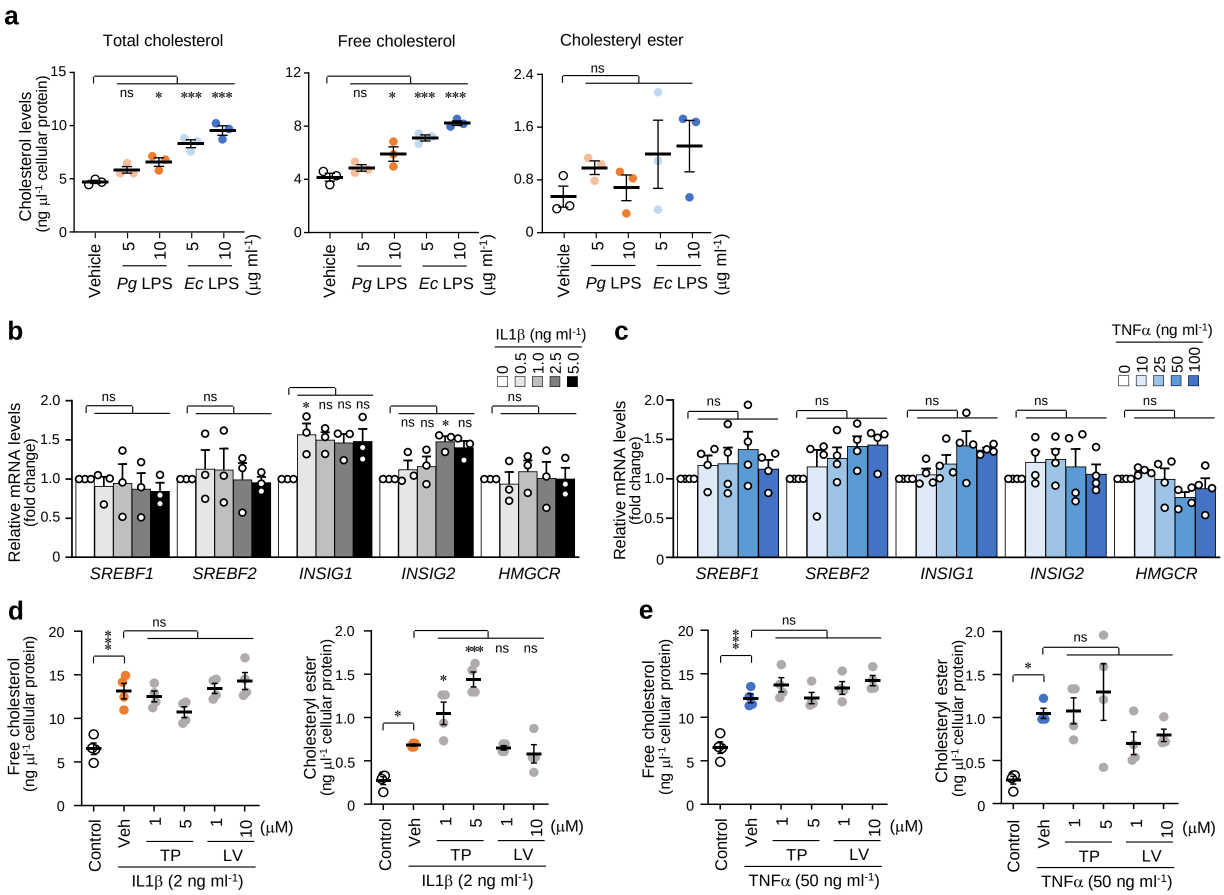

Supplementary Fig. 2. Expression of cholesterol biosynthesis-related genes and effects of cholesterol biosynthesis inhibitors. a Total cholesterol, free cholesterol, and cholesteryl ester levels in human GF treated with *Pg* LPS or *Ec* LPS for 24 h ($n = 3$). **b, c** qRT-PCR analysis of the indicated genes involved in cholesterol sensing and synthesis in human GF treated with IL1β (**b**) ($n = 3$) or TNFα (**c**) ($n = 4$) for 24 h. **d, e** Cellular levels of free cholesterol and cholesteryl ester in human GF treated with cholesterol synthesis inhibitors (triparanol [TP] and lovastatin [LV]) in the presence of IL1β (2 ng ml^{-1}) (**d**) or TNFα (50 ng ml^{-1}) (**e**) for 24 h ($n = 4$). *n* indicates the number of biologically independent samples. Values are presented as mean ± SEM with one-way ANOVA and Tukey's test. (*$P < 0.05$, **$P < 0.01$, ***$P < 0.001$).

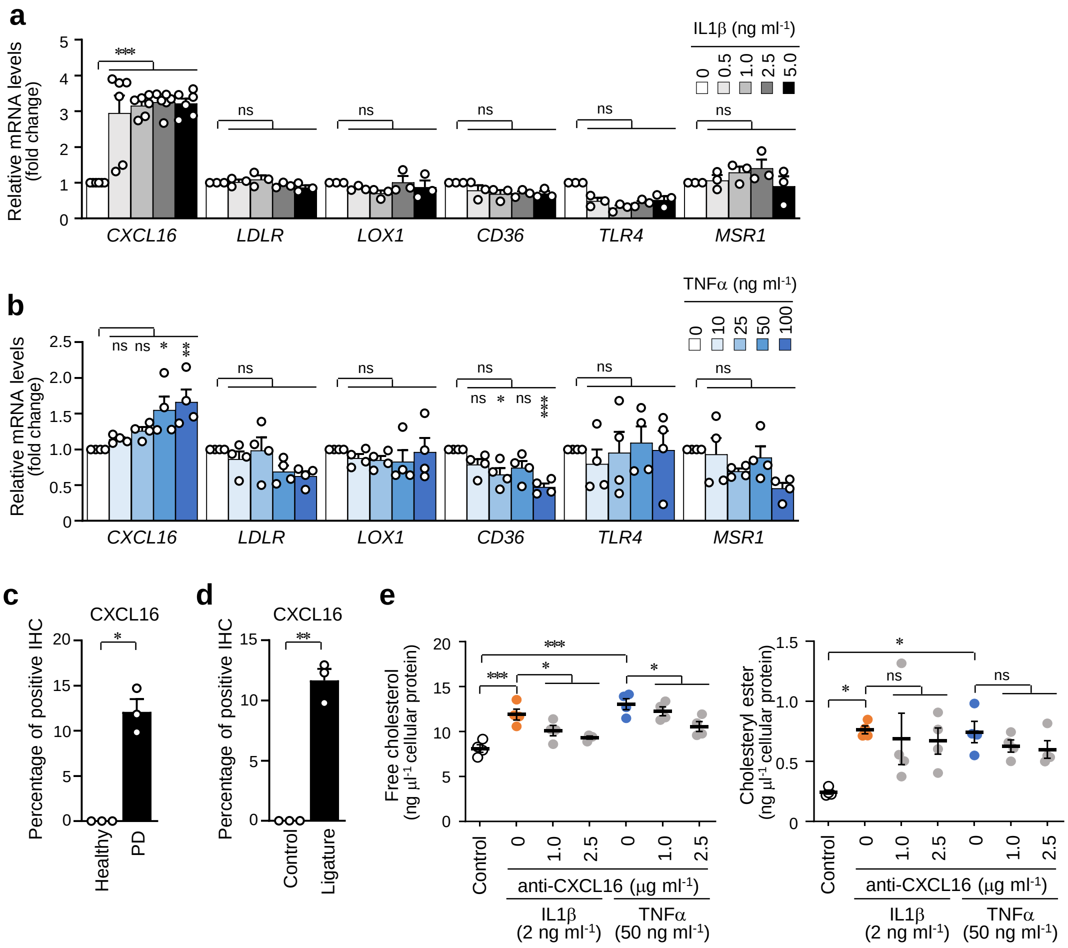

Supplemental Fig. 3. Upregulation of CXCL16 in inflamed periodontal cells and tissues and CXCL16-mediated increase in cellular cholesterol in GF. a, b Evaluation of mRNA levels of genes involved in cholesterol uptake in human GF treated with IL1β (**a**) or TNFα (**b**) for 24 h ($n \geq 3$). **c, d** Percentage of positive staining intensity of CXCL16 immunostaining of the gingiva from human patients with PD (**c**) and ligature-induced PD mice (**d**) ($n = 3$)**. e** Cellular levels of free cholesterol and cholesteryl ester in human GF treated with anti-CXCL16 blocking antibody in the presence of IL1β (2 ng ml^{-1}) or TNFα (50 ng ml^{-1}) for 24 h ($n = 4$). *n* indicates the number of biologically independent samples. Values are presented as mean ± SEM via two-tailed *t*-test (**c, d**) and one-way ANOVA and Tukey's test (**a, b, e**). (*$P < 0.05$, **$P < 0.01$, ***$P < 0.001$).

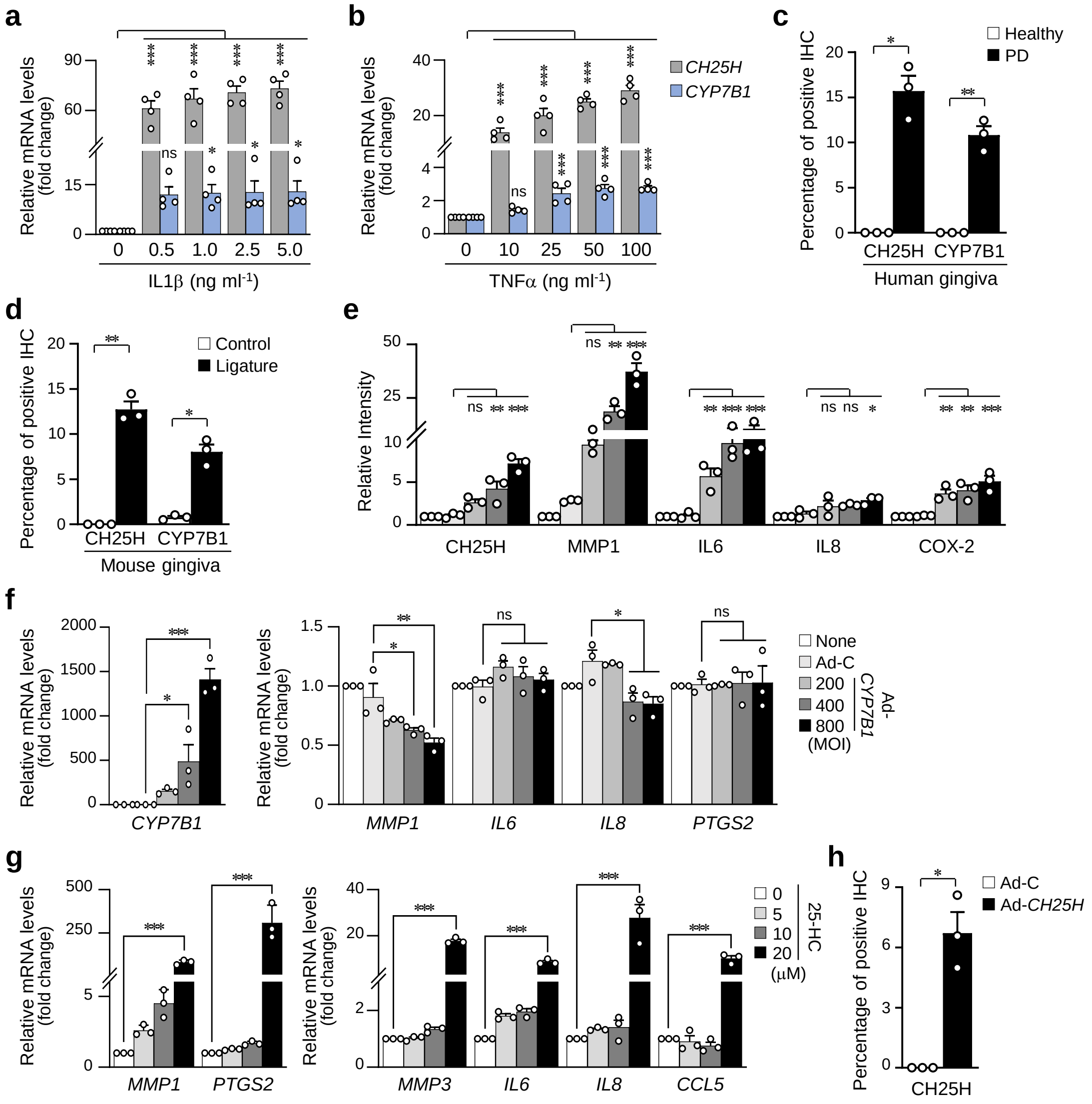

Supplementary Fig. 4. Upregulation of CH25H and CYP7B1 in PD-mimicking conditions and their functions in PD-associated catabolic gene expression. a, b mRNA levels of *CH25H* and *CYP7B1* in human GF treated with the indicated doses of IL1β or TNFα for 24 h (n = 4). **c, d** Graphical representation of percentage of positive staining intensity of CH25H and CYP7B1 immunostaining images in human (**c**) and mouse gingiva (**d**) (n = 3). **e** Relative intensities of CH25H, MMP1, IL6, IL8, and PTGS2 western blot images of human GF infected with *CH25H*-overexpressing adenovirus (Ad-*CH25H*; n = 3). **f** mRNA levels of *CYP7B1*, *MMP1*, *IL6*, *IL8*, and

PTGS2 in human GF infected with Ad-*CYP7B1* (n = 3). **g** mRNA levels in human GF treated with 25-HC for 48 h (n = 3). **h** Percentage of positive staining intensity of CH25H immunostaining images of mouse gingiva injected with an empty virus (Ad-C) or Ad-*Ch25h* (1×10^9 PFU per 5 μl) (n = 3). n indicates the number of biologically independent samples. Values are presented as mean ± SEM based on two-tailed t-test (**c, d, h**) and one way ANOVA and Tukey's test (**a, e, f, g**). (*P < 0.05, **P < 0.01, ***P < 0.001).

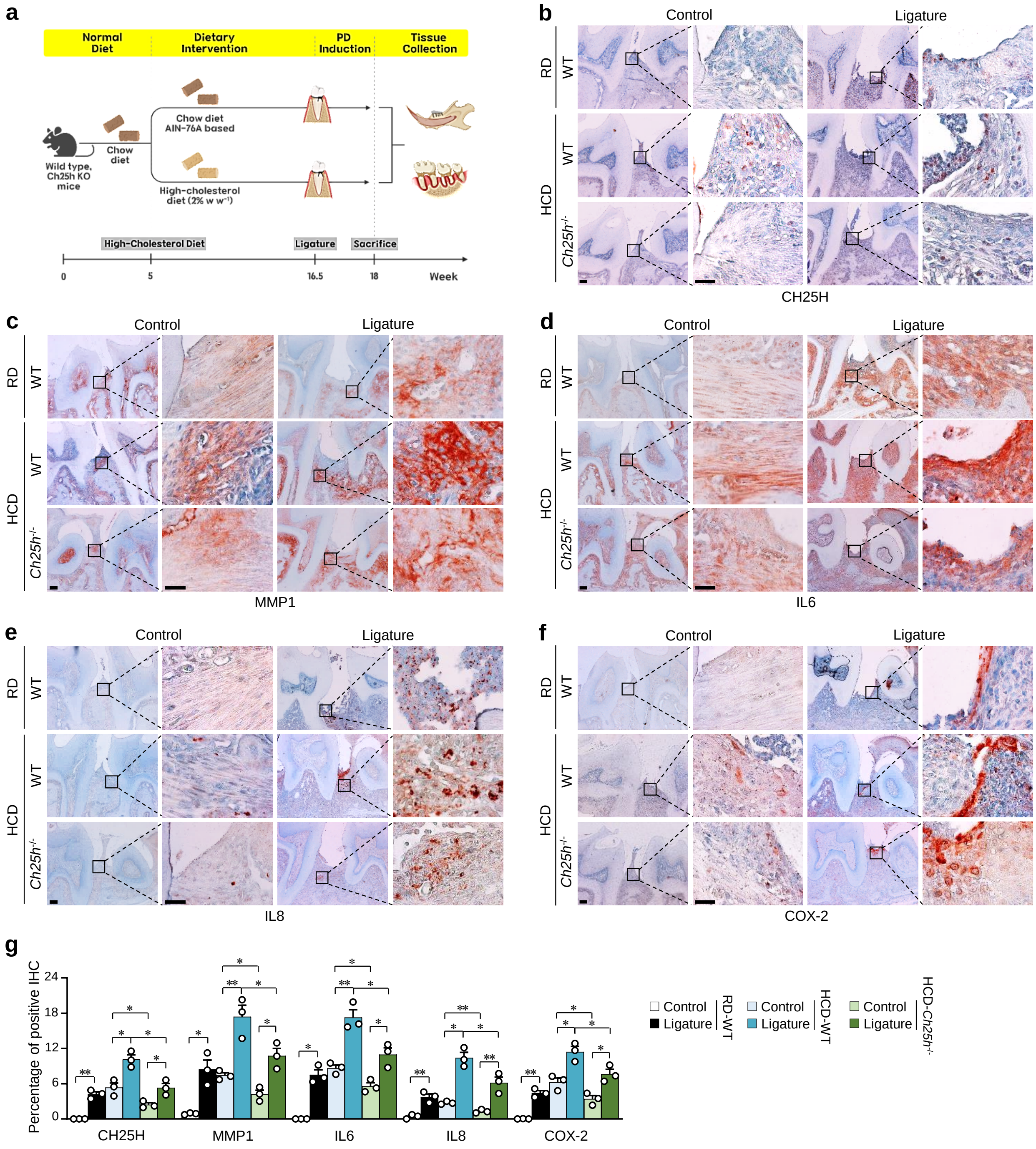

Supplementary Fig. 5. Alterations in PD-associated protein expression in *Ch25h*$^{-/-}$ mice. a Schematic diagram of experimental ligature-induced PD procedures of mice fed with RD or HCD for 13 weeks. Schematic diagram was created with BioRender.com. **b-f** Representative images of CH25H (**b**), MMP1 (**c**), IL6 (**d**), IL8 (**e**), and COX-2 (**f**) immunostaining from control and ligature-

induced PD gingiva of wild-type (WT) and *Ch25h* knockout (*Ch25h*$^{-/-}$) mice fed RD or HCD for 13 weeks. Scale bar, 100 μm (left), 25 μm (right). **g** Graphical representation of percentage of positive staining intensity of CH25H, MMP1, IL6, IL8, and COX-2 immunostaining images from control and ligature induced PD gingiva of wild-type (WT) and *Ch25h* knockout (*Ch25h*-/-) mice fed RD or HCD for 13 weeks (n = 3). Values are presented as mean ± SEM based on two-tailed *t*-test (**g**). (*P < 0.05, **P < 0.01, ***P < 0.001).

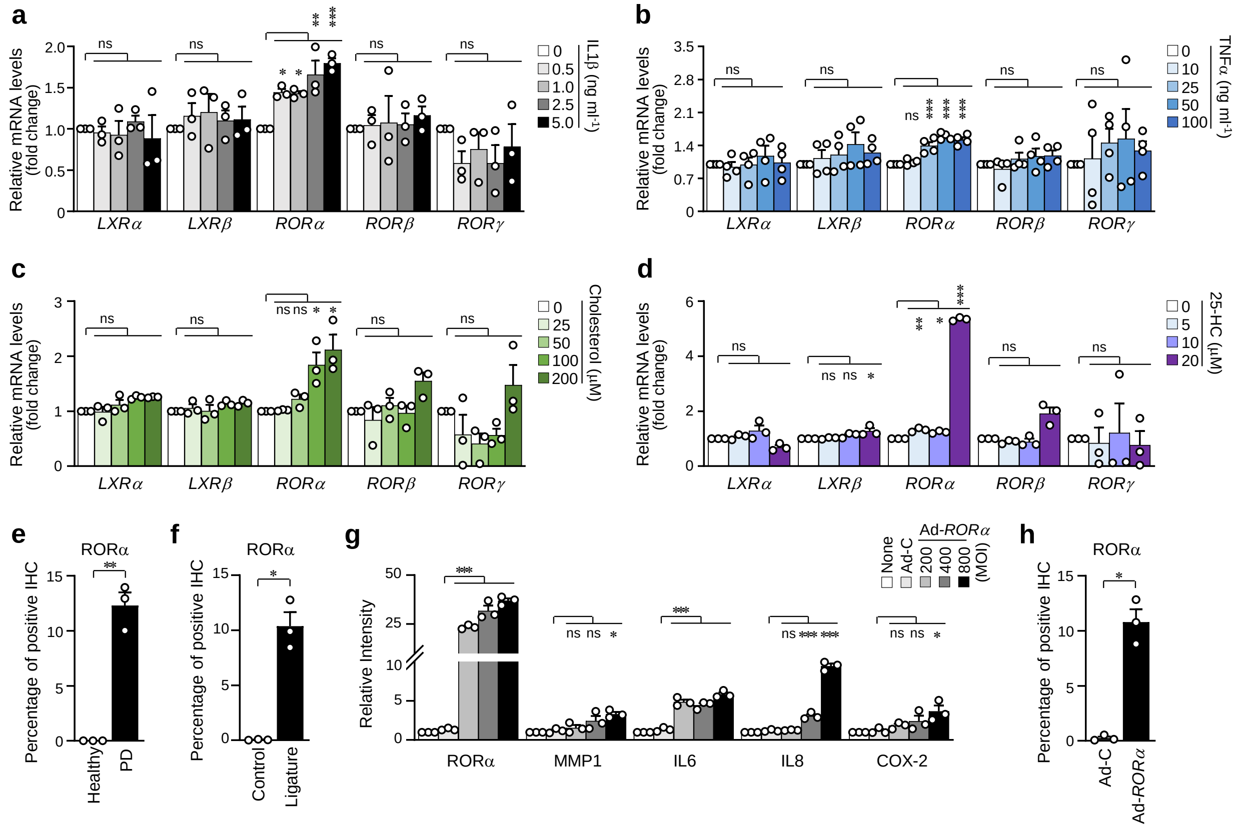

Supplementary Fig. 6. RORα expression is upregulated in PD-mimicking human GF. a-d qRT-PCR analysis of mRNA levels of nuclear receptors (*LXRα*, *LXRβ*, *RORα*, *RORβ*, *RORγ*) in human GF treated with the indicated doses of IL1β (**a**), TNFα (**b**), cholesterol (**c**), or 25-HC (**d**) for 24 h ($n \geq 3$). **e, f** Percentage of positive staining for *RORα* immunostaining in images from human (**e**) and mouse gingiva (**f**) (n = 3). **g** Relative intensities of *RORα*, MMP1, IL6, IL8, and PTGS2 western blot images in human GF infected with Ad-*RORα* for 48 h (n = 3). **h** Percentage of positive staining intensity of *RORα* immunostaining images in mouse gingiva injected with an empty virus (Ad-C) or Ad-*Rora* (1×10^9 PFU per 8 μl) (n = 3). n indicates the number of biologically independent samples. Values are presented as mean ± SEM based on two-tailed t-test (**e, f, h**) and one-way ANOVA and Tukey's test (**a, b, c, d, g**). (*$P < 0.05$, **$P < 0.01$, ***$P < 0.001$).

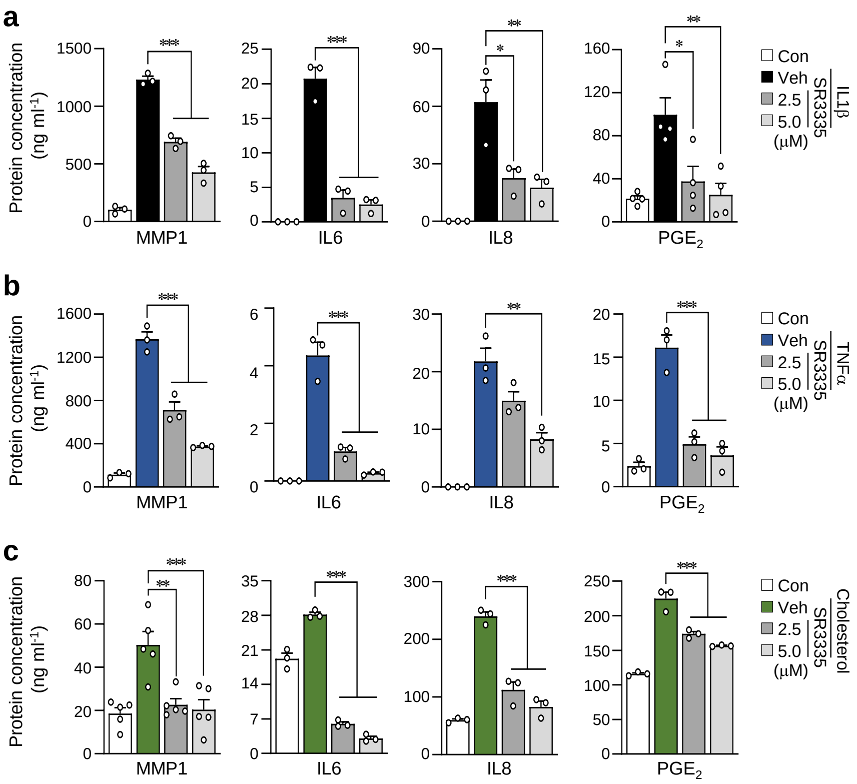

Supplemental Fig. 7. Blocking of RORα activation reduced the effects of IL1β, TNFα, and cholesterol on the expression of catabolic factors. a-c Secretion of MMP1, IL6, and IL8 and production of PGE_2 from human GF treated with the indicated concentrations of SR3335 in the presence of IL1β (2 ng ml^{-1}) (**a**), TNFα (50 ng ml^{-1}) (**b**), and cholesterol (200 μM) (**c**) for 24 h (*n* ≥ 3). *n* indicates the number of biologically independent samples. Values are presented as mean ± SEM based on one-way ANOVA and Tukey's test. (**P* < 0.05, ***P* < 0.01, ****P* < 0.001).

Supplementary Table. PCR primer sequence and PCR condition

Gene	Strand	Primer sequences	Size (bp)	AT (°C)	Origin
CCL5	S	5′-GGATCAAGACAGCACGTGGA-3′	421	60	Human
	AS	5′-CGGGTGGGGTAGGATAGTGA-3′			
CD36	S	5′-GAGAACTGTTATGGGGCTAT-3′	389	60	Human
	AS	5′-TTCAACTGGAGAGGCAAAGG-3′			
CH25H	S	5′-ACATCTGGCTTTCCGTGGAG-3′	139	60	Human
	AS	5′-TACGGAGCGAAGTTGCAGTT-3′			
CYP7B1	S	5′-TGCTGCAGTCAACAGGTCAA-3′	193	60	Human
	AS	5′-TTTCGCACACAGTAGTCCCC-3′			
CXCL16	S	5′-ATCTGCTTTTCACACTGGGT-3′	485	60	Human
	AS	5′-GAGGGGGAACCATCAGCAAA-3′			
GAPDH	S	5′-GGTGAAGGTCGGAGTCAACG-3′	327	60	Human
	AS	5′-CAAATGAGCCCCAGCCTTCT-3′			
HMGCR	S	5′-TTGGTGATGGGAGCTTGTTGT-3′	267	60	Human
	AS	5′-CGAGCCAGGCTTTCACTTCT-3′			
IL6	S	5′-AGGCTGGACTGCAGGAACTCCTTAAAG-3′	421	60	Human
	AS	5′-CCCTGAGAAAGGAGACATGTAACAAGAG-3′			
IL8	S	5′-CTCTCTTGGCAGCCTTCCTGATTTC-3′	254	60	Human
	AS	5′-AAACTTCTCCACAACCCTCTGCAC-3′			
INSIG1	S	5′-CATCTTTTCCTCCGCCTGGT-3′	247	60	Human
	AS	5′-ATGTCCACCAAAGGCCCAAA-3′			
INSIG2	S	5′-GCGGGGGATTTCTGGTAGG-3′	476	60	Human
	AS	5′-ACACCGCATTACACTGGACC-3′			
LDLR	S	5′-TGTTCCCACGTCTGCAATGA-3′	298	60	Human
	AS	5′-GGATGAGGCTGGTGTACTCG-3′			
LOX1	S	5′-GGCATGCAATTATCCCAGGTG-3′	283	60	Human
	AS	5′-TGCCAGATCCAGTCTTGCG-3′			
LXRα	S	5′-ACTGATGTTCCCACGGATGC-3′	338	60	Human
	AS	5′-CACAGTGTTAGCGAGGGCT-3′			
LXRβ	S	5′-CACAGTCACAGTCGCAGTCA-3′	208	60	Human
	AS	5′-TCGGAGAAGGAGCGTTTGTT-3′			
MMP1	S	5′-GGAGGGGATGCTCATTTTGATG-3′	541	60	Human
	AS	5′-TAGGGAAGCCAAAGGAGCTGT-3′			

MMP3	S	5′-AATCCTACTGTTGCTGTGCGTG-3′	238	60	Human
	AS	5′-CAGAGTGTCGGAGTCCAGCTTC-3′			
MSR1	S	5′-CCAATGAGAGGGATGAGAACTG-3′	129	60	Human
	AS	5′-GCTCAATGACAGCTTTGCTTC-3′			
PTGS2	S	5′-AATCCTTGCTGTTCCCACCCATG-3′	329	60	Human
	AS	5′-AAGGGAGTCGGGCAATCATCAGG-3′			
RORα	S	5′-GCAGATAACGTGGCAGACCT-3′	412	60	Human
	AS	5′-GCGATCCGCTGACATCAGTA-3′			
RORβ	S	5′-GGGTTATTACAACGTCGATTCCG-3′	358	60	Human
	AS	5′-CGTATTGGATGGCGTGAGTG-3′			
RORγ	S	5′-GCTCTGGGCCCTCATATTC-3′	315	60	Human
	AS	5′-TGGCATGTCTCCCTGTAGG-3′			
SREBF1	S	5′-CTGTTGGTGCTCGTCTCCTT-3′	214	60	Human
	AS	5′-CTACAAGCCAGGTCCAGGTG-3′			
SREBF2	S	5′-CAACTCCTATTCTTCAGCCCCG-3′	460	60	Human
	AS	5′-CGTCTGGATAGGGGTGGTGA-3′			
TLR4	S	5′-AACCTCCCCTTCTCAACCAA-3′	496	60	Human
	AS	5′-TCATAGGGTTCAGGGACAGGT-3′			

AT, annealing temperature; S, sense; AS, antisense.
